# Fabry disease; early diagnosis improves prognosis but diagnosis is often delayed

**DOI:** 10.15171/jnp.2017.22

**Published:** 2017-02-05

**Authors:** Jagadish Jamboti, Cynthia H Forrest

**Affiliations:** ^1^Department of Renal Medicine, Fremantle Hospital, Fremantle, Western Australia; ^2^University of Western Australia, Crawley, Perth, Australia; ^3^Department of Anatomical Pathology, Path West Laboratory Medicine, Fremantle Hospital, Fremantle, Western Australia

**Keywords:** Fabry disease, Alpha galactosidase, Electron microscopy, Stroke, Left ventricular hypertrophy, End stage kidney disease

## Abstract

**Background:**

Fabry disease (FD) is a rare X-linked deficiency of lysosomal enzyme alpha-galactosidase (AGAL) resulting in accumulation of globotriaosylceramide (Gb-3) in the cells, with protean manifestations. Major organs affected are the kidneys, heart and nervous system. The diagnosis of FD is often delayed by many years. Enzyme replacement started early might reverse the organ damage while delayed initiation may only stabilize the disease progression.

**Case Presentation:**

We describe a patient in whom involvement of different organs unfolded at different times and a detailed review of history by the clinician led to the diagnosis. The importance of electron microscopy (EM) of renal biopsy is highlighted.

**Conclusions:**

Patients with FD are often diagnosed late because the manifestations can be variable and spread over different time periods. Detailed history including family history and examining the renal biopsy by EM are crucial for early diagnosis.

**Implication for health policy/practice/research/medical education:**

The diagnosis of Fabry disease may be delayed because of its rare occurrence and varied manifestations. Appropriate diagnostic work up is highlighted in this case report.

## 1. Introduction

Fabry disease (FD) is a rare X-linked recessive disorder that leads to absent or low levels of the lysosomal enzyme alpha-galactosidase (AGAL) resulting in intracellular build-up of globotriaosylceramide (Gb-3) in various organs like skin, cornea, kidneys, central nervous system and heart, with protean manifestations (1,2). Diagnosis is established by low leucocyte AGAL levels. Awareness of the varied manifestations spread over a long time period with a high index of clinical suspicion are the key to detection of the disease. Electron microscopy (EM) of renal biopsy can reveal characteristic diagnostic findings. Enzyme replacement therapy can stabilize the clinical condition by preventing further progression of the disease.

## 2. Case Presentation

A 21-year-old man from a remote area in Western Australia was initially referred by the general practitioner (GP) to the renal service in 2001 with peripheral oedema and a family history of “nephritis” in his deceased grandfather. Serum creatinine was 150 μmol/L and urinary protein 1.5 g/24 h.

Renal biopsy at the time revealed focal segmental glomerulosclerosis (FSGS) with three out of six glomeruli revealing segmental sclerosis. The patient received corticosteroids with no improvement in proteinuria, therefore corticosteroids were tapered and ceased after three months.

**Figure 1:**
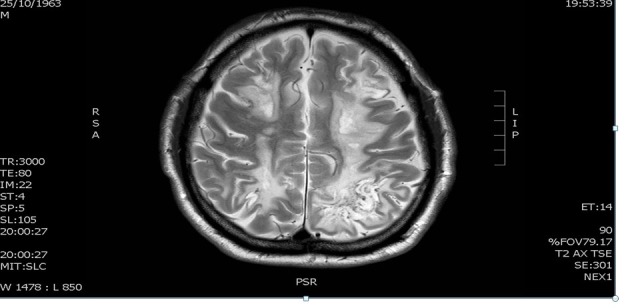
Showing diffuse White matter lesions on MRI of brain (T2 axial).

Ten years later, the patient was in stage 4 chronic kidney disease and had relocated to the Metropolitan area. Arteriovenous fistula was created in anticipation for future need for dialysis. Two months after relocating, the patient developed status epilepticus and required admission to the intensive care unit (ICU). Magnetic resonance imaging (MRI) of the brain revealed multifocal, bi-hemispherical white matter lesions. (Figure 1). Brain biopsy was performed which revealed minor and essentially nonspecific findings with no morphological changes diagnostic of an active vasculitis. In view of multiple cerebral infarcts and the well-recognized fact that a considerable proportion of cerebral biopsies performed in such cases could be non-diagnostic, the patient was treated for primary central nervous system (CNS) vasculitis. He received six doses of monthly IV cyclophosphamide followed by azathioprine for 12 months along with tapering dose of steroids. He had developed cortical blindness and required prolonged neurologic rehabilitation. He required ongoing hemodialysis, started during the above admission.

Twelve years after initial presentation, at 33 years of age, the patient was undergoing regular, uneventful maintenance hemodialysis and was assessed for suitability for renal transplant. He was found to have severe left ventricular hypertrophy on electrocardiogram (ECG) during renal transplant work up, despite having normal blood pressure and not being on any antihypertensive medications. Dobutamine stress test revealed severe dynamic left ventricular outflow obstruction and the possibility of familial hypertrophic cardiomyopathy was suggested. Coronary angiography was essentially normal.

A detailed assessment of the family history at this stage revealed that patient’s maternal grandfather had died in his thirties of renal failure presumably secondary to “chronic nephritis.” Patient’s mother was a single child of this person and had four children including one elder son aged 56 years who was apparently normal, and two younger daughters. The other family members had no symptoms to raise concerns. The patient is unmarried and has no children. With multi-system involvement (kidneys, CNS and heart) and positive family history, FD was suspected.

Blood sample was then sent to the national reference laboratory after explaining the suspected diagnosis of FD and its implications. Low leucocyte AGAL levels (0.2 n mol/min/mg protein [normal 0.7-3.3]) confirmed the diagnosis.

Molecular analysis of GLA gene revealed that the patient was hemizygous for p.N34T mutation resulting in the replacement of an asparagine residue with threonine residue, a mutation that has not been reported previously.

**Figure 2:**
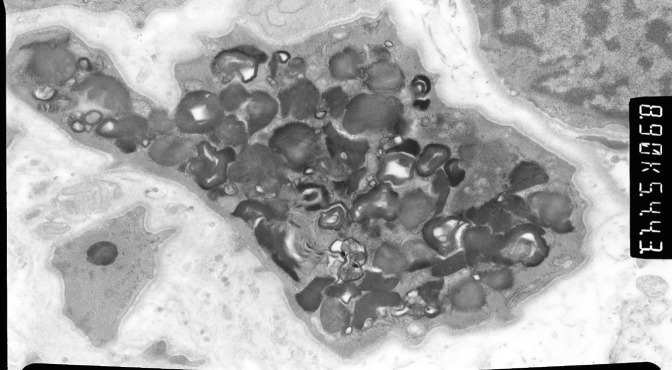
Higher power of lipid inclusions filling the cytoplasm. Many with a dense homogeneous appearance and a few with the more characteristic laminated (“zebra stripes”) appearance.

The initial renal biopsy was retrieved and reviewed by EM at this point and revealed the characteristic laminated electron-dense lipid deposits in endothelial cells and macrophages giving rise to zebra stripe and myelin body appearances (Figure 2).

The patient was started on Fabrazyme (Alfa galactosidase beta; 1 mg/kg fortnightly) infusions to be continued for life. One year follow up revealed no further seizures (on anti-epileptic therapy), transient ischemic attack (TIA) or stroke, while continuing on maintenance haemodialysis.

## 3. Discussion

Fabry disease (Anderson-Fabry disease) is an X-linked deficiency of lysosomal enzyme AGAL leading to Gb-3 accumulation in cells. The characteristic skin lesions (angiokeratoma) were described independently by William Anderson in England and Johannes Fabry in Germany in 1898. The causative enzyme deficiency was described in the 1960s. Enzyme replacement therapy was introduced in 2001 (3).

Birth prevalence of FD was previously estimated at 1:117000 in Australia (4). A more recent newborn screening by assaying the alpha-Gal A activity in blood spots from 37104 consecutive Italian male neonates revealed AGAL deficiency in 12, suggesting a much higher incidence of 1 in 3100. Enzyme-deficient infants were re-tested and “doubly screened-positive” infants and their relatives were diagnostically confirmed by enzyme and mutation analyses (5).

Our patient has been detected with a novel gene mutation, being p.N34T hemi zygote, adding to the more than 300 mutations of AGAL already reported.

With abnormal AGAL being unable to break down Gb-3 effectively, Gb-3 builds up in the body’s cells, particularly cells lining blood vessels in the skin and cells in the kidneys, heart, and nervous system. The progressive accumulation of Gb-3 in various organs leads to the varied signs and symptoms of FD.

In the CNS, acroparesthesias (pain crises) due to small pain fibre involvement, reduced or absent sweating due to autonomic neuropathy and stroke have been described. In the Fabry registry of 2446 patients, 138 patients suffered stroke; the median age at first stroke was 39.0 years in males and 45.7 years in females. The cause of stroke was ischemic in 85% of cases while 15% of strokes were hemorrhagic, attributed to hypertension (7). Dolichoectasia (elongation and dilatation) of basilar artery has been described as a classical finding leading to Pulvinar sign hyper-intensity of posterior thalamus on traction-1 imaging by MRI. Not all the patients with stroke demonstrate Gb-3 accumulation in the major or microscopic blood vessels of the brain. The pathogenesis of Fabry vasculopathy remains poorly understood but is associated with abnormal functional control of the vessel secondary to endothelial dysfunction, cerebral hyper-perfusion and a pro-thrombotic state with likely increased production of reactive oxygen species (7,8).

By middle age, most patients develop renal insufficiency leading to end-stage renal disease, as well as cardiac and cerebrovascular disease. Major causes of morbidity and mortality are usually attributed to renal (proteinuria and progressive renal failure), cardiac (hypertrophic cardiomyopathy) and cerebrovascular complications. (9) The initial renal pathological changes are seen in podocytes due to Gb-3 accumulation. Progressive glomerular injury is associated with mesangial widening and ultimately with segmental and global glomerulosclerosis. FD nephropathy is associated with only a moderate prevalence of hypertension, when compared to other chronic kidney diseases (10)

The diagnosis of FD is often delayed by a decade or more from the initial presentation. As per Australian data the median age at diagnosis is 28.6 years, although in hindsight, many of the patients would have been symptomatic for more than a decade. Early diagnosis and enzyme replacement therapy might limit the severity of the disease manifestations with improved outcomes (3).

Alternative therapeutic approaches, including small molecule chaperone therapy, are currently being explored (11).

Awareness of the protean manifestations occurring over a long period of time and the importance of eliciting detailed family history in establishing the diagnosis, are highlighted in this case report. Arning et al suggested a questionnaire to help pick up Fabry patients (12). EM has a significant role in diagnosing FD, demonstrating diagnostic laminated electron-dense lipid deposits in various cells. In the absence of medication history with drugs like amiodarone and chloroquine which can cause lysosomal injury, the characteristic EM findings of laminated electron-dense lipid deposits in endothelial cells and macrophages leading to ‘zebra stripe’ and ‘myelin body’ appearances are diagnostic of FD.

## 4. Conclusions

Patients with FD are often diagnosed late because the manifestations can involve different organ systems at different time periods. Detailed history including family history and examining the renal biopsy by EM aid in early diagnosis. Early initiation of enzyme replacement therapy might limit the progression and improve the outcomes.

## Acknowledgements

Prof. Mark Thomas, consultant nephrologist and I/C Fabry Clinic, Royal Perth Hospital, Wellington Street, Perth Western Australia and Dr. Michael Fietz, head of national reference laboratory, Adelaide, South Australia.

## Authors’ contribution

All authors contributed equally in the preparation of the case report.

## Conflicts of interest

The authors declare no conflict of interest.

## Funding/Support

There was no source of funding for this publication.
